# Continuous Glucose Monitoring Data Sharing in Older Adults With Type 1 Diabetes: Pilot Intervention Study

**DOI:** 10.2196/35687

**Published:** 2022-03-16

**Authors:** Nancy A Allen, Michelle L Litchman, James Chamberlain, Ernest G Grigorian, Eli Iacob, Cynthia A Berg

**Affiliations:** 1 College of Nursing University of Utah Salt Lake City, UT United States; 2 St Mark’s Diabetes Center St Mark's Hospital Salt Lake City, UT United States; 3 College of Social and Behavioral Science University of Utah Salt Lake City, UT United States

**Keywords:** older adults, type 1 diabetes, caregiver, CGM, data sharing, mobile phone

## Abstract

**Background:**

Family members or friends (care partners [CPs]) of older adults with type 1 diabetes (T1DM) regularly become part of the diabetes care team, but they often lack knowledge about how to become involved to prevent hypo- and hyperglycemia. Continuous glucose monitoring (CGM) allows a person with diabetes to see their glucose levels continuously and to receive predictive alerts. A smartphone data-sharing app called the Follow app allows the person with diabetes to share continuous glucose numbers with others and to receive predictive alerts of impending hypo- and hyperglycemia. However, there are barriers to sharing this continuous glucose level data with CPs.

**Objective:**

This study aimed to address the barriers to sharing CGM data. Our objective was to examine the feasibility of using CGM with the Follow app and a data-sharing intervention called SHARE *plus* in older adults with T1DM and their CPs. SHARE *plus* includes dyadic communication strategies, problem-solving strategies, and action planning to facilitate CGM data sharing.

**Methods:**

Older adults with T1DM (n=20) and their CPs (n=20) received the SHARE *plus* intervention at baseline. People with diabetes wore the CGM for 12 weeks while sharing their glucose data using the Follow app with CPs. Feasibility data were analyzed using descriptive statistics.

**Results:**

The SHARE *plus* intervention was feasible and was associated with high self-reported satisfaction for people with diabetes and their CPs as well as high adherence to CGM (mean 96%, SD 6.8%). Broad improvements were shown in the diabetes-related quality of life through the use of CGM in people with diabetes and their CPs. Although the majority of people with diabetes (11/20, 55%) were willing to share hyperglycemia data, several chose not to. The majority of people with diabetes (14/20, 70%) were willing to talk about glucose numbers with a CP.

**Conclusions:**

Older adults with T1DM and their CPs identified having someone else aware of glucose levels and working together with a partner on diabetes self-management as positive aspects of the use of the SHARE *plus* intervention. Clinicians can use these results to provide data sharing coaching in older adults and their CPs.

## Introduction

An estimated 1.59 million individuals have type 1 diabetes (T1DM) in the United States [1], with a growing number of adults living into late adulthood, as life expectancy has increased by 15 years in the past 70 years [2]. Severe hypoglycemia occurs most frequently in adults >50 years old with T1DM and results in seizure or loss of consciousness [3]. The risk for severe hypoglycemia is markedly increased in older adults because of reduced awareness of hypoglycemic warning symptoms, reduced hormonal counter-regulatory response, and changes in dexterity, visual acuity, fine motor skills, cognitive function, depression, and anxiety that may prevent affected individuals from taking corrective actions [4]. These age-related changes result in increased complications related to hypo- and hyperglycemia, including myocardial infarction, cerebrovascular accidents, dementia, dementia-related falls, fractures, and sudden death [4,5]. Moreover, hyperglycemia increases the risk of dehydration, electrolyte imbalances, urinary incontinence, dizziness, and falls [6]. Family members and friends of older adults with T1DM regularly become part of the diabetes care team. However, these care partners (CPs) often lack knowledge about when or how to become more involved to prevent hypo- and hyperglycemia.

Since Medicare began covering continuous glucose monitoring (CGM), access to CGM has increased among older adults with T1DM and it has shown some efficacy at reducing risk of hypo- and hyperglycemia in these individuals. The Diamond and WISDM (Wireless Intervention for Seniors with Diabetes Mellitus) trials [7,8] demonstrated that CGM in older adults improves time in range (70-180 mg/dL), reduces glucose variability, hypoglycemia, and improves hemoglobin A_1c_ in comparison to blood glucose monitoring. Although time in range improved significantly in both trials at 6 months, time in range only increased by 93-100 minutes per day in the majority of CGM users. Mixed results were seen for participant satisfaction and diabetes-related quality of life (DQOL) across the 2 trials. Though this evidence supports the use of CGM in older adults, limited research is focused on using CGM with CPs and how it may affect a person with diabetes.

Several CGM systems have apps that allow a CP to see CGM glucose levels continuously and receive alerts and a hypoglycemia alarm. Dexcom has a mobile app called Follow that allows CPs to access glucose data for people with diabetes [9]. Although no studies have assessed the experience of using CGM with the Follow app in older adults and their CPs, data suggest that adults and their CPs may benefit from assistance in knowing how to be involved for optimal diabetes management [10,11]. However, there are barriers to using Follow, such as the need for knowledge on smartphone technology, difficulties setting up the sharing features [10], and challenges in communication between people with diabetes and CPs that reflect difference in expectations with regard to CP involvement [10]. People with diabetes and their CPs indicate that expectations of CP involvement in diabetes management frequently differ between them. People with diabetes often regard diabetes as “their own illness,” whereas their spouses view the condition as “shared” [12,13]. However, when people with diabetes and their spouses both appraise T1DM as shared, collaboration and support are more frequent [10,13]. Specifically, there is increased self-care and self-efficacy in people with diabetes because of increased perceptions of emotional support and decreased critical communication from the CP [10,14]. Notably, older adults are more likely to appraise diabetes as shared [11].

Our prior research with adults and CGM reveals several barriers to the use of Follow among adults, namely the need for knowledge on smartphone technology, difficulties setting up the sharing features, and challenges in dyadic communication that reflect people with diabetes and partners’ different expectations regarding family involvement [10]. SHARE *plus* addresses these barriers by providing instruction to use the technology, training in dyadic communication and problem solving, and a data-sharing action plan developed with people with diabetes and their CPs. However, there is a lack of effective interventions to support people with diabetes and their families in adopting and successfully using CGM with the Follow app.

To address the current gaps in CGM data sharing among older adults with T1DM, this study examined the feasibility of a CGM with a Follow app intervention, SHARE *plus*, among people with diabetes and their CPs.

## Methods

### Study Design

A 1-group experimental design was chosen to determine if there was interest and adherence to the intervention and to identify the components of the intervention that need refinement. This study was approved by the University of Utah Institutional Review Board (00114642). Participants signed an institutional review board informed consent.

Participants were recruited from an academic endocrinology clinic and an internal medicine/diabetologist office in Utah and included people with diabetes and their CPs (spouse, adult child, friend; henceforth called dyads when both are referenced). People with diabetes were included if they were ≥60 years, were diagnosed as having T1DM, had normal or mild cognitive impairment (MCI; Montreal Cognitive Assessment [MoCA] score 18-26) [15], were unfamiliar with using personal CGM with a Follow app, had hemoglobin A_1c_ values of 6%-12% within the last 6 months, were able to read and write English, had a CP willing to participate, and owned a smartphone compatible with the Dexcom G6 data sharing system. People with diabetes with or without an insulin pump were included. People with diabetes were excluded if they had the following: an estimated life expectancy of less than 1 year; unstable recent cardiovascular disease, significant malignancy, or other conditions resulting in physical decline; a history of visual impairment that would hinder their ability to perform all study procedures safely; or a history of psychiatric, psychological, or psychosocial issues that could limit adherence to required study tasks. CPs were included if they were identified by the person with diabetes, willing to participate in the CGM education sessions and CGM, ≥18 years of age, and owned a smartphone compatible with the Dexcom G6 CGM data sharing system. CP exclusion criteria included no self-report of cognitive impairment or dementia or other medical conditions that the investigator determined would make it inappropriate or unsafe to fulfill a CP’s role.

From the pool of potential participants (N=123), 20 (16.2%) people with diabetes and their CPs (20, 16.2%) met the recruitment criteria. The remaining participants did not meet the recruitment criteria for the following reasons: could not be reached by phone or letter (65/123, 52.8%), had no time or interest in research (16/123, 13%), had no CP (6/123, 4.8%), had an incompatible phone (7/123, 5.7%), had cancer (2/123, 1.6%), had Parkinson disease (1/123, 0.8%), had moderate or severe dementia (3/123, 2.4%), and exhibited delusional behavior (2/123, 1.6%).

### Procedures

Following a screening visit and participant enrollment, data were collected at baseline and at 3 months. After baseline data were collected, people with diabetes and CPs received basic CGM training using technical manuals and components of a Dexcom G6 training video that was adapted for use in older adults with CPs by a trained research assistant. At that same visit, people with diabetes and their CPs received the SHARE *plus* intervention. People with diabetes were asked to wear the CGM with the Follow app for 3 months and share CGM data using the intervention strategies. They continued to follow up with their health care provider to manage any changes in their treatment plan. Final study data were collected at a 3-month follow-up visit, followed by a one-on-one interview with the person with diabetes and the CP. All visits were conducted in the home of the person with diabetes to minimize difficulty associated with navigating a large academic health science center.

### Intervention

Dyads participated in an interactive CGM with data sharing intervention (Textbox 1). The intervention included the following behavioral components: communication strategies, problem-solving strategies, and action planning. SHARE *plus* included evidence-based strategies such as motivational interviewing, problem solving, self-efficacy enhancement, and action planning [16-18].

**Components of the data sharing intervention SHARE plus.**

1. Communication strategies

- Communication strategies around using real-time continuous glucose monitoring with the Follow app. People with diabetes were asked about their willingness to talk about glucose numbers (hypo- and hyperglycemia). The objective of this discussion was to determine what glucose information the person with diabetes was comfortable sharing.

2. Problem-solving strategies

- Barriers to sharing glucose levels were identified and discussed (eg, glucose levels are private, people with diabetes do not want to be judged).
- Problem-solving around expectations and length of waiting time before the care partner should contact the person with diabetes for a concerning glucose level and the preferred mode that the care partner uses to contact the person with diabetes (eg, phone call, SMS text messaging, email) were identified. Dyads engaged in a discussion and problem solving around alarms for the Follow app on the person with diabetes and care partner’s smartphone to determine an agreeable strategy. The objective of this step was to guide the dyad on how to manage real-time continuous glucose monitoring expectations and how to incorporate SHARE into their lives.
- People with diabetes identified how they wanted the care partner involved (when and how to respond, troubleshooting). Care partners were asked how they feel about this type of communication and if it is acceptable. The objective of this discussion was to explore supportive and unsupportive conversation strategies between dyads.

3. Action plan

- Communication plan in writing that includes how to give feedback, length of waiting time, communication mode.
- Set alarms with people with diabetes and care partners (each can have different alarms).
- Written responsibility and frequency of monitoring glucose levels for people with diabetes and care partners.
- Actions to take for severe low blood sugar, chest pain and symptoms of heart attack or stroke, etc.

### Measures

#### Quantitative Feasibility Measures

The following data were examined: retention, reasons for study discontinuation, feasibility (appointment attendance, length of all sessions, number of unscheduled appointments for extra assistance, number of telephone calls for the person with diabetes or CP support), and implementation (percentage of protocol completion, barriers).

#### Clinical and Person With Diabetes-Reported Outcomes

Demographics and cognitive status (MoCA) [15] were assessed at baseline. Adherence data were obtained via Dexcom Clarity, a secure online program that captured the amount of wear time from the CGM data of the person with diabetes [19]. Glucose data were obtained via Dexcom Clarity [19]. Data included percentage of time in range (70-180 mg/dL), hypoglycemic range (<60 mg/dL), hyperglycemic range (>250 mg/dL), and glycemic variability coefficient value.

DQOL using CGM was measured at 12 weeks using a 15-item instrument with 3 subscales: perceived control (α=.88), hypoglycemia safety (α=.84), and interpersonal support (α=.75) [20]. Individuals were asked to indicate how each item has changed since they started using CGM with the Follow app. Responses were rated on a 5-point scale (scores 1 to 5), with higher scores indicating improvement [20].

#### Qualitative Satisfaction Data

Dyads were asked 4 questions on what they liked and did not like about sharing CGM data with their CP, what recommendations they have for others who share CGM data, and what recommendations they have for intervention improvements.

### Analytic Plan

Data were analyzed using descriptive statistics with means and SDs for continuous variables (summary scores) and frequency counts and percentages for categorical data. A content analysis was conducted on the open-ended satisfaction questions. The satisfaction responses were read word for word and then coded. Next, the coded data were categorized and summarized.

## Results

### Feasibility, Demographics, and Clinical Characteristics

People with diabetes (n=20) had a mean age of 70 (SD 5) years and diabetes duration of 31 (SD 18.30) years, and the majority were married (13/20, 65%), White individuals (18/20, 90%), and male (11/20, 55%). The majority wore an insulin pump (11/20, 55%) and had previously used CGM but were naïve to using the Follow app (11/20, 55%), while 9/20 (45%) had never used CGM. There were 8/20 (40%) participants that required extra assistance or support with initial CGM use; 4 (50%) of these participants were naïve to wearing CGM and 4 (50%) had previous experience with CGM. The people with diabetes had a variety of comorbid conditions (Table 1). CPs (n=20) had a mean age of 57 (SD 17) years, and the majority were White individuals (19/20, 95%) and female (13/20, 65%). The retention rate was 95% over 3 months. One participant discontinued the study—a person with diabetes who reported that the alarms were disruptive, loud, and too frequent (alarm settings chosen by the participant were 90 mg/dL lows and 180 mg/dL highs) and that sensor adhesion was poor (did not use the waterproof film offered). This participant also had a MoCA score of 24, which was consistent with MCI.

**Table 1:** Demographics for people with diabetes and their care partners.

Demographics							RT-CGM^a^ user (n=20)							CP^b^ (n=20)
**Age (years)**														
	Mean (SD)									70.45 (4.90)				56.6 (16.75)
	Median (IQR)									69 (66-73.8)				62.5 (41.5-69.8)
**Gender, n (%)**														
	Male										11 (55)		7 (35)	
	Female										9 (45)		13 (65)	
**Ethnicity, n (%)**														
	Hispanic or Latino								2 (10)				1 (5)	
	Not Hispanic or Latino					18 (90)							19 (95)	
**Race, n (%)**														
		White					17 (85)							19 (95)
	African American							0 (0)					0 (0)	
	Native American/Alaskan/Pacific Native					2 (10)							0 (0)	
	Other					1 (5)							1 (5)	
**Marital status, n (%)**														
	Single											2 (10)	5 (25)	
	Married											13 (65)	13 (65)	
	Divorced											5 (25)	2 (10)	
**Highest education, n (%)**														
			High school or less				1 (5)							2 (10)
			Technical/associate/some college				6 (30)							7 (35)
			Bachelor’s degree				6 (30)							7 (35)
			Master’s degree				4 (20)							3 (15)
			Doctoral degree				3 (15)							1 (5)
**Employment status, n (%)**														
			Full-time				6 (30)							9 (45)
			Part-time				2 (10)							3 (15)
			Retired				11 (55)							6 (30)
			Unemployed				0 (0)							2 (10)
			With a disability				1 (5)							0 (0)
**Household income, n (%)**														
				$34,999 or less			3 (15)							4 (20)
				$35,000 to $49,999			2 (10)							3 (15)
				$50,000 to $99,999			5 (25)							10 (50)
				$100,000 to $149,999			6 (30)							3 (15)
				Declined to state income			4 (20)							0 (0)
Diabetes duration, mean (SD)							30.9 (18.27)							
**Comorbid conditions, n (%)**														
					Hypothyroidism		10 (50)							N/A^c^
					Hypertension		7 (35)							N/A
					Dyslipidemia		5 (25)							N/A
					Gastrointestinal disease		5 (25)							N/A
					Retinopathy		4 (20)							N/A
					Neuropathy		4 (20)							N/A
					Depression		4 (20)							N/A
					Stroke		3 (15)							N/A
					Myocardial infarction		2 (10)							N/A
					Nephropathy		2 (10)							N/A

^a^RT-CGM: real-time continuous glucose monitoring.

^b^CP: care partner.

^c^N/A: not applicable.

The MoCA screening test showed that 45% (9/20) of people with diabetes had a MoCA score <26 (range 20-29), indicating MCI. Those with MCI had a mean age of 71.4 (SD 6) years and median diabetes duration of 30 (IQR 7-45) years, and the majority were White individuals (8/9, 89%) and male (6/9, 67%). Those without MCI had a mean age of 69.6 (SD 4) and median diabetes duration of 30 (IQR 20-45) years, and the majority were White individuals (9/11, 81%) and female (6/11, 56%). The majority of people with diabetes without MCI wore an insulin pump (8/11, 73%), had previously used CGM but were naïve to using the Follow app (8/11, 73%), and had Medicare insurance coverage (8/11, 73%). There were no differences in CGM glycemic data or the results of the satisfaction survey between those who completed the assessment and had a MoCA score in the MCI range and those who did not have a score in this range.

#### Feasibility Results

The initial SHARE *plus* appointment averaged approximately 1 hour (19 appointments; mean time 67.65 minutes, SD 29.12 minutes). Unscheduled appointments for extra assistance were for the following: sensor failure (6/20, 30%), transmitter failure (1/20, 5%), and connectivity issue that was solved by disconnecting and waiting for 30 seconds (1/20, 5%). Sensor and transmitter difficulties were attributed to storage at a high temperature. All participants received 100% of the intervention. People with diabetes wore CGM for the majority of the 12-week study (mean adherence 96%, SD 6.8%).

All participants were willing to share their hypoglycemia data, but only 55% (11/20) were willing to share their hyperglycemia data (Table 2). The interventionist was trained to give participants a choice about sharing their hyperglycemia data. The primary reasons cited for declining to set alarms for hyperglycemia were “do not care about hyperglycemia because the person with diabetes can handle that on their own without problems” and “already understand highs and do not need help with this.” There were no reported complications from this decision to use the Follow app for hypoglycemia only. The majority of dyads set the Follow app alarms higher than the alarms for people with diabetes.

**Table 2:** SHARE plus intervention data.

SHARE intervention components		Responses, n (%)
Agreement to share hypoglycemia data (yes)		20 (100)
**Agreement to share hyperglycemia data**		
	Yes	11 (55)
	Maybe	1 (0.5)
**Agreement by people with diabetes to share hyperglycemia data by age (years)**		
	65-69	4 (20)
	70-74	4 (20)
	75-79	2 (10)
	80-84	2 (10)
People with diabetes willing to talk about glucose numbers with a CP^a^		14 (70)
**Helpful comments to support a person with diabetes**		
	“Are you okay”	11 (55)
	“Your sugar is low, what do you need”	4 (20)
	“What can I do to help”	2 (10)
	“Your blood sugar is low, you need to eat”	3 (15)
**Alarms on the Follow app**		
	Same alarms for CPs and people with diabetes	6 (30)
	Higher alarms for CPs	5 (25)
	CP turns alarms off for highs	9 (45)
**Waiting time to contact the person with diabetes about low alarm (min)**		
	0 (immediately)	6 (30)
	5	8 (40)
	10	2 (10)
	15	2 (10)
	20	1 (0.5)
**Contact mode**		
	Phone call	12 (60)
	SMS text messaging	6 (30)
	Phone call and SMS text messaging	2 (10)
	No data	1 (0.5)
**Action to take if no reply to low alarm**		
	Call friend/family	10 (50)
	Come to the home of the person with diabetes	6 (30)
	Call emergency medical services	4 (20)

^a^CP: care partner.

#### Clinical and Participant Reported Outcomes

People with diabetes spent the majority (median 62%) of their time in range (70-180 mg/dL) and had minimal time spent in the hypoglycemic range (median <1%). The SHARE *plus* intervention was not targeted at improving glucose levels, and there were no observed trends in the CGM data showing significant differences between baseline and 12 weeks. Hemoglobin A_1c_ data were not obtained because of limited funding, but the glucose management indicator obtained from CGM data was hemoglobin A_1c_ of 8.3%.

At the end of the third month, DQOL using CGM was measured. Broad improvement was noted for the perceived control domain (77% people with diabetes, 75% CP), hypoglycemia safety (74% people with diabetes, 63% CP), and interpersonal support (63% people with diabetes, 63% CP).

#### Qualitative Feasibility Measures

People with diabetes and their CPs reported high satisfaction with SHARE *plus*, with more likes than dislikes reported (Table 3-5).

**Table 3:** Satisfaction survey responses for people with diabetes.

Question and theme for people with diabetes		Value, n (%)
**Likes about RT-CGM^a^ data sharing**		
	Having someone else aware of glucose levels	8 (40)
	Having a partner work together	4 (20)
	Receiving help from care partner	4 (20)
	Partner can notice challenges	2 (10)
**Dislikes about RT-CGM data sharing**		
	Nothing	10 (50)
	Partner nagging or overreacting	3 (15)
**Recommendations for other people like you for RT-CGM with data sharing**		
	Highly recommend	11 (55)
	Take time to understand diabetes	1 (5)
**Recommended intervention improvements**		
	Nothing	9 (45)
	More education	4 (20)

^a^RT-CGM: real-time continuous glucose monitoring.

**Table 4:** Satisfaction survey responses for care partners.

Question and theme for care partner		Value, n (%)
**Likes about RT-CGM^a^ data sharing**		
	Constantly being able to see the glucose numbers	13 (65)
	Peace of mind knowing partner is alright	7 (35)
	Work as a team	3 (15)
**Dislikes about RT-CGM data sharing**		
	Nothing	12 (60)
	Not always accurate	2 (10)
	Scared with seeing lows	2 (10)
**Recommendations for other people like you for RT-CGM with data sharing**		
	Highly recommend	10 (50)
	Important to have a good relationship	3 (15)
	Have good communication established	2 (10)
**Recommended intervention improvements**		
	Nothing	13 (65)
	More education	6 (30)

^a^RT-CGM: real-time continuous glucose monitoring.

**Table 5:** Exemplar satisfaction quotes from people with diabetes and their care partners.

Question	Exemplar quotes from people with diabetes	Exemplar quotes from care partners
Likes about RT-CGM^a^ data sharing	“That if I am having a low blood sugar someone else is aware and can help if I need it” “Made us both aware of my situation and allowed us to work together on my progress and challenges” “He saved me by calling when I had a very low blood sugar” “She sees how challenging it is to maintain good control”	“I liked being able to have instant access to her numbers” “It was comforting to know where his blood sugars were” “It is very helpful and allows us all as a family to suggest treatment decisions”
Dislikes about RT CGM data sharing	“I usually knew what was going on, was a little irritating to have him remind me” “They over-reacted”	“Some inconsistencies between [meter] and CGM and variable times losing contact with sensor data” “I got scared a few times when he had lows and maybe I worried about him more than when I didn’t know”
Recommendations for other people like you for RT-CGM with data sharing	“I felt freedom and constant knowledge of glucose. Do it! Do it! Freedom” “Be patient and just know that your partner is looking out for you” “Diabetes is a roller coaster experience, it will take time to learn how to deal with it!”	“Highly recommend the CGM and shared data, has helped the family dynamics (i.e., reducing anxiety and constant stress of asking ___ to check his blood sugars” “My husband is exceptional with no temper. It might be hard for some people if they didn’t have the right kind of relationship” “As long as there is already good communication and the [person with diabetes] is willing to take responsibility rather than making you their ‘blood sugar police,’ I think it can be great”
Recommended intervention improvements	“I need to know more about adjusting alarm sounds for highs” “A bit more training on the computer program that stores the results?” “The clarity apps are helpful, and produce a big picture of the complications of the disease, but they do not help much when I want to know how many units of insulin I need to drop or increase the reading by ‘x’ units”	“Follow up every week” “….Talk about the [CGM] data over time” “Remember older people might forget certain things over time like” calibrating” CGM with [meter] blood glucose readings” “Could have used additional written instructions on how to install a new transmitter to the phone”

^a^RT-CGM: real-time continuous glucose monitoring.

The majority of people with diabetes liked the CP support they received from data sharing. However, 3/20 (15%) individuals reported that CPs nagged or overreacted. The CPs liked the ability to see the data, which gave them peace of mind. A total of 2/20 (10%) CPs reported concerns about CGM accuracy. With regard to education, 4/20 (20%) people with diabetes and 6/20 (30%) CPs wanted more education. The majority of dyads recommended CGM with data sharing, and a few CPs cited the importance of having a good relationship and good communication skills.

There were 9/20 (45%) people with diabetes who were new to using CGM. Of these 9, 5 (56%) requested more education on insulin adjustments, changing sensors and alarms, and tracking events and alarms. Of the 11/20 (55%) people with diabetes who had previously worn the CGM device, only 3 (27%) requested more education on using Dexcom Clarity and adjusting alarm sounds for high glucose readings.

Of the 20 dyads, 18 (90%) were cohabiting. Of the remaining 2/20 (10%) CPs, 1 was a son and the other was a friend. The friend CP only had positive feedback on the satisfaction survey, but the son CP did not like “getting alarms at all hours” and wanted more education on how to “review past data and be able to do comparisons to see if things are getting worse or better.”

Our key recommendations based on these feasibility data are in Textbox 2. These recommendations include increasing the acceptability around data sharing and hyperglycemia data sharing, decreasing nagging and overreaching behaviors, increasing diabetes education, and implementing strategies to monitor SHARE *plus* behaviors in real time.

**Key recommendations.**

1. Overarching recommendations

- Increase acceptability of data sharing
- Increase willingness to share hyperglycemia and hypoglycemia data
- Improve communication to decrease nagging and overreaching
- Increase dyad diabetes education around diabetes self-management using CGM with Follow
- Monitor SHARE plus behaviors in real time

2. Specific strategies

- Coach dyads on the concept of sharing diabetes vs viewing diabetes as only the person with diabetes
- Intensify the case for using the Follow app for hyperglycemia (teamwork, support, working together)
- Provide 3-4 diabetes education sessions to address intensified problem-solving and communication strategies and dyadic self-management—management of hyper- and hypoglycemia
- Measure SHARE plus agreement changes and how conflicts were addressed over time using ecologic momentary assessment methods

## Discussion

The SHARE *plus* intervention comprised communication strategies, problem solving, and an action plan and was feasible and associated with high satisfaction among dyads. Our results show broad improvements in DQOL across dyads in the following domains: perceived control of diabetes, hypoglycemia safety, and interpersonal support. Although the majority of people with diabetes were willing to share hyperglycemia data and discuss glucose levels with their CPs, some chose not to. Lastly, people with diabetes identified having someone else aware of glucose levels and working together with a partner on diabetes self-management as positive aspects of using CGM with SHARE *plus*.

Similar to our study, both the WISDM and Diamond trials showed an average wear time of 6 days a week or greater during the study period [7,8]. While several people with diabetes chose not to share their hyperglycemia data or discuss their glucose numbers with their CPs at baseline, the SHARE *plus* intervention in its present form provided little discussion around these topics. These agreements may have changed over time and were not measured in this study.

Several key recommendations for future studies include intensifying the case for hyperglycemia data sharing (teamwork, support, working together). Additional recommendations include more diabetes education sessions that include communication and problem-solving strategies, glucose management, and the use of CGM software to track glucose trends. In another study, our team found that spouses understand how to assist with some diabetes-related recommendations, such as supporting hypoglycemia [11]. However, they may not understand how to manage microadjustments around glucose levels, which older adults with diabetes may need assistance with as they age [11]. However, some people with diabetes may never want to involve their CPs in diabetes management. Further studies are needed to examine changes in the attitudes of people with diabetes with intensification of SHARE *plus* combined with diabetes education. Additionally, further research is needed to track the number of diabetes management interventions (changes in diet and insulin dosing) resulting from use of the Follow app and whether there is a difference in the behavioral and glucose outcomes between those who agree to engage more with their CPs and those who do not. Ecological momentary assessment allows researchers to track behavior in real time and may be a method to capture many SHARE *plus*–related behaviors, including conflicts and how sharing agreements change over time [21].

There are benefits and disadvantages of CP involvement in CGM data sharing. The positive aspects of CGM data sharing identified by people with diabetes included having someone else aware of glucose levels, working together with a partner on diabetes self-management, and receiving help from a partner. This type of collaboration likely occurs as the person with diabetes and their partners see T1DM as shared [13]. Further research is needed to assess if people with diabetes and their CPs appraise T1DM as shared in the context of SHARE *plus*; this is associated with better self-care and self-efficacy in people with diabetes because of increased perceptions of greater emotional support and decreased critical communication from CPs [14].

CPs often walk the line between trying to be supportive and being overbearing [11]. Very few individuals in our study reported “nagging” or “overreacting” to data sharing. It is unclear if our study’s reported benefits are related to CGM alone or the SHARE *plus* intervention. However, dyads receiving SHARE *plus* rated their interpersonal support much higher (63%) than those in a CGM study without data sharing (37%) [22]. Yet, this higher rating of interpersonal support in our study may be attributed to differences between older adults in our study and middle-aged individuals in the comparison study [22]. A fully powered randomized controlled trial is needed to demonstrate the effects of the SHARE *plus* intervention versus CGM alone and the difference in interpersonal support.

SHARE *plus* is promising as it addresses gaps related to CGM data sharing in older adults. However, our results indicate that further strategies are needed to improve SHARE *plus*. Future iterations of SHARE *plus* should include diabetes education specific to glucose management and the use of Dexcom Clarity for glucose pattern management. Additional communication strategies are also needed to reduce the nagging and overreacting associated with data sharing that people with diabetes reported. Lastly, it is unknown how frequently CPs viewed the Follow app. It is possible that the frequency of viewing the app may be related to a person with diabetes’ experience of more or less critical behaviors. Future studies are needed to elucidate this possible relationship. Opportunities exist for clinicians working with CGM to encourage conversations with CPs that are positive, helpful, and acceptable to people with diabetes.

This study is not without limitations. This study intended to examine the feasibility and therefore was not powered to detect significant changes across variables, and findings should be interpreted cautiously. A total of 16 people with diabetes declined to participate in this study because of lack of time or disinterest in research. This may be attributed to a disinterest in using CGM or involving CPs in diabetes management. However, there has been growing interest in CGM since CGM was covered by Medicare in 2017. Further exploration of people with diabetes’ disinterest in participating in a dyadic study is needed. This study lacked racial and ethnic diversity. Moreover, participants were highly educated. However, this feasibility study’s initial results suggest the need for a larger study with a more diverse sample and an assessment of technology literacy. Willingness of a person with diabetes to share hyperglycemia data or discuss glucose data was only assessed at baseline. This initial reaction may have changed either positively or negatively over time. Future studies should evaluate this willingness over time.

The results are promising in that they show that older adults with T1DM are open to sharing their glucose data with CPs and that CPs report benefits with assistance in communication and problem-solving strategies as to how to collaborate most effectively with people with diabetes. The benefits of such an intervention may become more important as older adults age and experience complications from lifelong diabetes, especially cognitive challenges that make self-management more challenging. The potential benefits of SHARE *plus* are consistent with those of dyadic approaches to chronic illness management that may enhance not only self-care but the quality of life for both people with diabetes and their CPs.

## Abbreviations

CGM: continuous glucose monitoring
CP: care partner
DQOL: diabetes-related quality of life
MCI: mild cognitive impairment
MoCA: Montreal Cognitive Assessment
T1DM: type 1 diabetes
WISDM: Wireless Intervention for Seniors with Diabetes Mellitus
